# Grain nitrogen content and productivity of rice and maize under variable doses of fertilizer nitrogen

**DOI:** 10.1016/j.heliyon.2023.e17321

**Published:** 2023-06-19

**Authors:** Surajit Mondal, Rakesh Kumar, Janki Sharan Mishra, Anchal Dass, Sanjeev Kumar, Kumar Varun Vijay, Manisha Kumari, Sana Raza Khan, Vinod Kumar Singh

**Affiliations:** a Division of Crop Research, ICAR Research Complex for Eastern Region, Patna 800 014, Bihar, India; b Division of Agronomy, ICAR Indian Agricultural Research Institute, New Delhi 110 012, India

**Keywords:** Chlorophyll content, Grain N content, N uptake, NUE, Rice-maize, Soil total N

## Abstract

The rice-maize system is a dominant cropping system of south Asia and consumes a considerable amount of fertilizer. The indiscriminate use of fertilizer particularly nitrogen (N) is degrading the soil health and polluting the environment. Lower N-use efficiency is a major problem and needs to be improved for higher yield, lower cost of cultivation and better environment. The grain quality is also altered by the N-application as N is a major constituent of protein. Studies on the effect of N-application on grain N-content is still lacking. We hypothesised that optimization of N application would result in economising N dose, improving yield and NUE and improving grain quality. Under that context, a field experiment was conducted with different doses of fertilizer N for rice and maize. Fertilizer N was applied at the rate of 0, 40, 80, 120, 160, 200 and 240 kg ha^−1^ (N0–N240). An increase in grain yield was observed up to 80 and 160 kg ha^−1^ for rice and maize, respectively. The N content of grain increased with N rates and a significant increase was noted in N200 (1.42%) being at par with N240 (1.49%) but significantly higher than others by 13–32%. With an increase of each kilogram of N, the grain N content increased by 14 and 20 μg (microgram) for rice and maize, respectively. The leaf N content registered a decreasing trend with the progress of the crop growth for both rice and maize. The agronomic efficiency (AE) of N initially increased with an increase in the rate of fertilizer N followed by a decrease with higher doses of N. Unlike the AE, the partial factor productivity (PFP) of N decreased gradually with an increase in the rate of fertilizer N. The chlorophyll content of flag leaves also registered an increasing trend with an increasing rate of fertilizer N. On the surface soil (0–15 cm), the treatments which received lower (N0, N40) and higher (N240) fertilizer N recorded a comparatively higher total soil N than other treatments. The mean NUE was 0.42 and 0.75 for rice and maize, respectively. The study suggests an economic fertilizer N rate of 165 and 167 kg N ha^−1^, for rice and maize, respectively. It also concludes that the grain N content can be altered by N-application rates though more research is needed.

## 1 Introduction

Nitrogen (N), the most limiting essential plant nutrient of soil, is a prerequisite for modern highly productive agriculture [1,2]. The main source of nitrogen for crop production is synthetic fertilizer and without that global food production will be sufficient enough to feed only half of the population [3]. The global population which is projected to be increased by 2–3 billion by 2050 will substantially increase the demand for N fertilizer [4,5]. According to an estimate, about 67% of the fertilizer N is eventually transformed into the non-reactive atmospheric N_2_ and the rest 33% form reactive species and have transient effects on the global ecosystem [2,6].

Judicious N management can be instrumental in the nourishment of a growing population and for higher profitability. A lower N-use means lower productivity, malnutrition, and soil degradation while higher N-use has a larger impact on the ecosystem. Hence a balance must be maintained to among crop productivity, environment and cost of cultivation. The production systems should follow the pathway to achieve sustainable development goals [2,7]. The nitrogen use efficiency (NUE), the fraction of applied N harvested as a product, has been proposed as an index for assessing the progress of achieving sustainable environment goals [8]. Increasing NUE in agriculture is crucial for achieving triple challenges of food security, ecosystem degradation and climate change [9,10]. Hence, improvement in N-use efficiency is imperative for sustainable agriculture [11]. Available literature suggests a very low NUE of 31% for irrigated rice in Asia while maize has somewhat a higher NUE of 37% [12]. The average global NUE needs to be increased from ∼0.4 to ∼0.7 to achieve food security and environmental stewardship in 2050 [9].

Rice-maize double cropping system is recently emerging as a major cropping system in South Asia and is cultivated in about 3.5 M ha area in Asia [13]. This production system has high nutrient demand due to its high grain yield and extracts a considerable amount of nutrients from the soil. Rice and maize use about 40% of total N used globally [14]. Based on global data, the estimated fertilizer N recovery by above-ground biomass of rice is only about 46% [15] while the rest is lost through major pathways of NH_3_ volatilization and nitrification-denitrification [16]. Therefore, proper nutrient management strategies must be developed for the optimization of fertilizer use by lowering loss and increasing efficiency [17]. Raun and Johnson [18] have estimated a savings of USD 2.3 billion in terms of annual fertilizer N cost for every 1% increase in NUE while Kant et al. [19] reported USD 1.1 billion in savings for a similar increase in NUE.

Modern agriculture should focus on increasing crop yield with better nutritional quality for achieving food and nutritional security. Improving nutrient use efficiency in general and N in particular, is necessary to increase the productivity and quality of crops, reduce input cost for fertilizer and improve soil, water and environment quality [20]. The present recommended dose of N for rice and maize practised in India is very old. Farmers profusely use N fertilizer in the form of urea without considering the crop requirements. An excessive use of N fertilizer or imbalance in fertilization leads to lower profitability. Due to continuous mining of nutrients, soil degradation, depletion in organic matter content and micronutrients and adoption of high-yielding nutrient-responsive hybrids, there is a need to relook at the old recommendation to find out site-specific and need-based nutrient management strategies to reduce the N losses and improve NUE.

We hypothesised that optimization of N application would result in economising N dose, improving yield and NUE. Our main objective was to examine the effect of variable doses of fertilizer N on grain N content or protein content. The outcome of the present investigation would help in a better understanding of the N dynamics in rice and maize and will help in designing better N management strategies to improve the NUE. In that context, a well-planned field experiment was laid out to monitor the nitrogen uptake pattern of rice and maize throughout the growing period under different doses of fertilizer N application. The plant growth parameters, yield, grain N content and soil N status were monitored. The optimization of nitrogen fertilizer for higher yield with better grain quality could be instrumental for achieving food and nutritional security.

## 2 Materials and methods

### 2.1 Site description and treatment details

An experiment was conducted at the Indian Council of Agricultural Research (ICAR) – Research Centre for Eastern Region, Patna, Bihar, India with different N doses. The site is located at 25.591°N, 85.084°E. The location has a subtropical monsoonal climate with an annual average rainfall of 1167 mm. About 75–80% of the annual rain is received in July–September. The soil of the site is neutral in pH (7.5), non-saline and has a clay loam texture with sand, silt, and clay percentage of 25.9, 39.6 and 34.5%, respectively. The initial total soil N concentration was 0.091, 0.064 and 0.055% in 0–15, 15–30 and 30–45 cm soil layer, respectively.

Rice crop was grown from June to October while maize crop was raised from November–April. The experiment was laid out in a quasi-experimental design to minimize the effect of N fertilization from one treatment to another treatment. Seven field blocks were created, and polythene lining was used up to a depth of 20 cm to minimize the nutrient movement between treatments. Each block was further divided into five plots of dimension of 5 m × 4.5 m (22.5 m^2^). The net area of the entire experiment was 787.5 m^2^. The treatments of N doses were imposed in ascending order (lowest to highest). The rates of fertilizer N were 0, 40, 80, 120, 160 and 240 kg ha^−1^.

### 2.2 Crop management

In rice, 21 days old seedlings were transplanted in puddled plots in the third week of July 2021 at 20 and 15 cm row to row and plant to plant spacing, respectively. In maize, hybrid seeds were dibbled in a conventionally tilled plot at 50 and 25 cm row and plant spacing, respectively in the last week of November 2021. Five rice (*Swarna Shreya, Swarna Shakti, Swarna Samridhi, Swarna Sukha Dhan* and *Swarna Unnat Dhan*) and five hybrid maize cultivars (*Kanak 51, DMRH 1301, DKC 9188, DKC 9165* and *DKC 9081*) were used. The fertilizer dose was 60–60 and 75–60 kg (P_2_O_5_–K_2_O) for rice and maize, respectively while nitrogen fertilizer was applied as per treatments. Diammonium phosphate, single super phosphate, urea and muriate of potash were used as sources of nitrogen, phosphorous and potassium. The entire amounts of phosphorous and potassium were applied as basal at the time of planting while each 1/3rd of N was broadcasted at the transplanting, tillering and panicle initiation stages. In the case of maize, N application stages were seeding, knee-height stage and tasselling. For weed control, a preemergence herbicide namely pretilachlor @ 0.75 kg a.i. ha^−1^ was applied 2 days after transplanting (DAT) and one manual hand weeding was done at 45 DAT. In maize, two manual hand weeding were done at 40 and 75 days after sowing. Flood irrigation was uniformly applied in all the treatments as per the requirement.

### 2.3 Total N and C determination

The leaf, grain and straw samples were finely ground and used to determine the total N concentration. The dry combustion method was followed, and samples were combusted at 950 °C in a CHNS analyzer (Unicube, Elementar, GmbH). The total N concentration was then multiplied by the respective grain or straw yield to get the total uptake.

### 2.4 Yield and system productivity

The rice and maize were harvested at maturity at ground level and total biomass was noted after drying. After threshing (rice) and shelling (maize), the grain yield was noted. Moisture correction was applied for both straw and grain yield. To compare the system productivity among treatments, the maize grain yield was converted to rice equivalent yield (REY) [Eq. (1)]. The system productivity was calculated as the sum of rice grain yield and REY of maize for each treatment [Eq. (2)].

(1) REY of maize = [(Maize grain yield × MSP of maize)/(MSP of rice)]

(2) System productivity = Rice grain yield + REY of maize

where, MSP- Minimum support price of Govt. of India.

### 2.5 Soil sampling and analysis

After maize harvest, core soil samples of 5 cm height and 5.3 cm diameter as well as bulk samples were collected from 0–15, 15–30 and 30–45 cm soil layers. Collected soil samples were air-dried, ground, passed through a 2 mm sieve, and stored for further analysis. Available N was determined by the KMnO_4_ method. Soil cores were oven dried at 100 °C till constant weight and used for determination of soil bulk density.

### 2.6 Plant growth parameters

The plant growth parameters were monitored to detect changes due to differential fertilizer N application. The normalized difference vegetation index (NDVI) was measured by a GreenSeeker (Trimble). The instrument has an active light source and measures the reflected lights to quantify the greenness. At each stage, tillers of randomly selected four hills were counted and noted. Similarly, one hill was harvested at each sampling stage and biomass was measured after oven drying at 65 °C. The flag leaf of the same plant was ground and used for total N determination. The chlorophyll contents of the flag leaf and the topmost fully expanded leaf of rice and maize, respectively were measured spectrophotometrically by Hiscox and Israelstam method [21].

### 2.7 Nitrogen use efficiency and N-balance

Nitrogen use efficiency was evaluated in terms of two indexes i.e., agronomic use efficiency [Eq. (3)] and partial factor productivity [Eq. (4)] and measured by the following formulae:

(3) Agronomic use efficiency (kg yield kg^−1^ N) = (Yield of treatment -Yield of control)/Fertilizer N applied

(4) Partial factor productivity = Yield/Fertilizer N applied

where control means treatment with no added fertilizer.

### 2.8 Statistical analysis

All data were subjected to statistical analysis and the analysis of variance (ANOVA) was conducted as per the general linear model. The means of parameters were separated by Tukey's honestly significant difference test at P < 0.05. The principal component analysis and correlation matrix were done in Minitab (ver. 17.1.0). MS Excel was used for regression analysis and preparation of graphs.

## 3 Results

### 3.1 Effect of different doses of N on grain and leaf N content

**Fig. 1:**
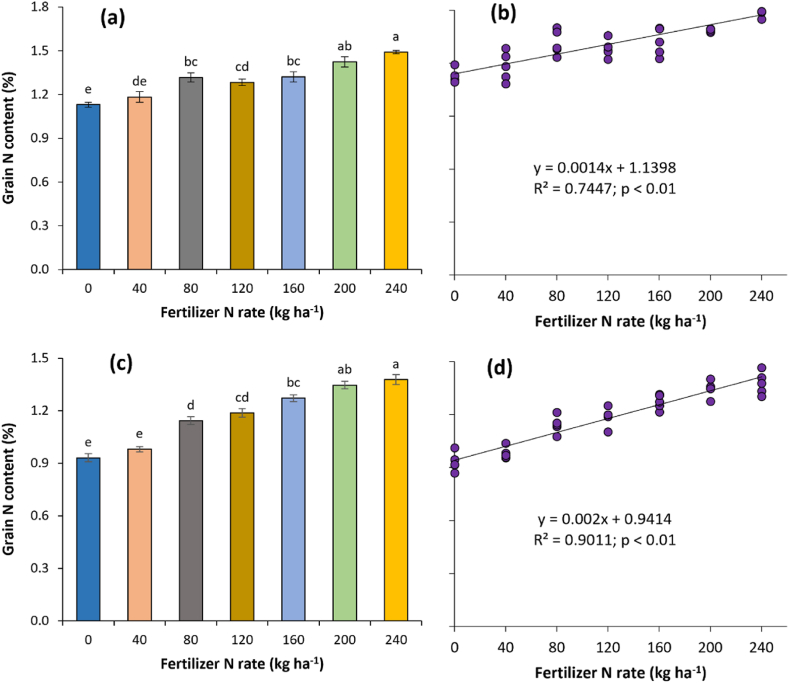
Grain N content of rice (a, b) and maize (c, d) as affected by different doses of fertilizer N. The vertical bars indicate ±standard error of means. Bars followed by at least one same lowercase letter are not significantly different at p < 0.05 by Tukey's Honestly Significant (HSD) test.

The highest rice grain N content of 1.49% was noted for N240 treatment which was at par with N200 (1.42%) but significantly higher than others by 13–32% (p < 0.05) (Fig. 1a). A similar trend of grain N content was also noted for maize, and higher N rates (N200 and N240) recorded greater grain N content (9–48%) than 0–160 kg N ha^−1^ (Fig. 1c). The regression suggested an increase of grain N content by 14 and 20 μg (microgram) for each kilogram of N for rice and maize, respectively (Fig. 1b and d). The coefficient of determination or predictability of grain nitrogen content based on fertilizer N was stronger for maize (R^2^ = 0.90; p < 0.01) than rice (R^2^ = 0.74; p < 0.01). Irrespective of crops, an increase in leaf N content was noted with increasing fertilizer N application (Table 1). Higher N application recorded 81, 72 and 35% increase in leaf N content over control (no N) at 39, 49 and 59 DAT, respectively while the same was 56, 83, 151 and 69% at 55, 63, 73 and 82 DAT for maize. The leaf N content registered a decreasing trend with the progress of the crop growth for both rice and maize.

**Table 1:** Leaf N content due to different doses of fertilizer n application in rice and maize. Means followed by SE of means. Mean values followed by at least one lowercase letter are not significantly different at p < 0.05 by Tukey's HSD test. DAT: Days after transplanting; DAS: Days after sowing.

Treatments	Leaf N content (%)			
	Rice			
	27 DAT	39 DAT	49 DAT	59 DAT
N0^a^	2.31ab ± 0.07	1.53e ± 0.10	1.10c ± 0.03	1.36c ± 0.04
N40	2.33ab ± 0.05	1.79de ± 0.05	1.40b ± 0.11	1.48bc ± 0.01
N80	2.45a ± 0.03	2.29bc ± 0.09	1.46b ± 0.06	1.52bc ± 0.09
N120	2.47a ± 0.08	2.10cd ± 0.05	1.52b ± 0.07	1.58abc ± 0.08
N160	2.15b ± 0.06	2.52ab ± 0.09	1.58b ± 0.05	1.62abc ± 0.08
N200	2.47a ± 0.04	2.66a ± 0.06	1.90a ± 0.04	1.68ab ± 0.05
N240	2.33ab ± 0.06	2.77a ± 0.08	1.89a ± 0.05	1.84a ± 0.06
Mean	2.36	2.23	1.55	1.58
	**Maize**			
	**55 DAS**	**63 DAS**	**73 DAS**	**82 DAS**
N0	2.72b ± 0.25	2.28c ± 0.22	1.36c ± 0.07	2.12c ± 0.04
N40	2.59b ± 0.14	3.03b ± 0.23	1.65c ± 0.03	2.12c ± 0.04
N80	3.73a ± 0.14	3.55ab ± 0.08	2.48b ± 0.16	2.85b ± 0.09
N120	4.05a ± 0.10	3.59ab ± 0.11	2.82b ± 0.18	3.02b ± 0.08
N160	3.81a ± 0.10	3.83a ± 0.08	2.91ab ± 0.11	3.20ab ± 0.05
N200	4.05a ± 0.05	3.61ab ± 0.10	2.96ab ± 0.08	3.31ab ± 0.12
N240	4.25a ± 0.13	4.18a ± 0.24	3.41a ± 0.14	3.59a ± 0.24
Mean	3.60	3.44	2.51	2.89

a The number after N indicates kg of fertilizer N applied per ha. DAT: Days after transplanting; DAS: Days after sowing.

### 3.2 Crop productivity as affected by variable doses of N

**Fig. 2:**
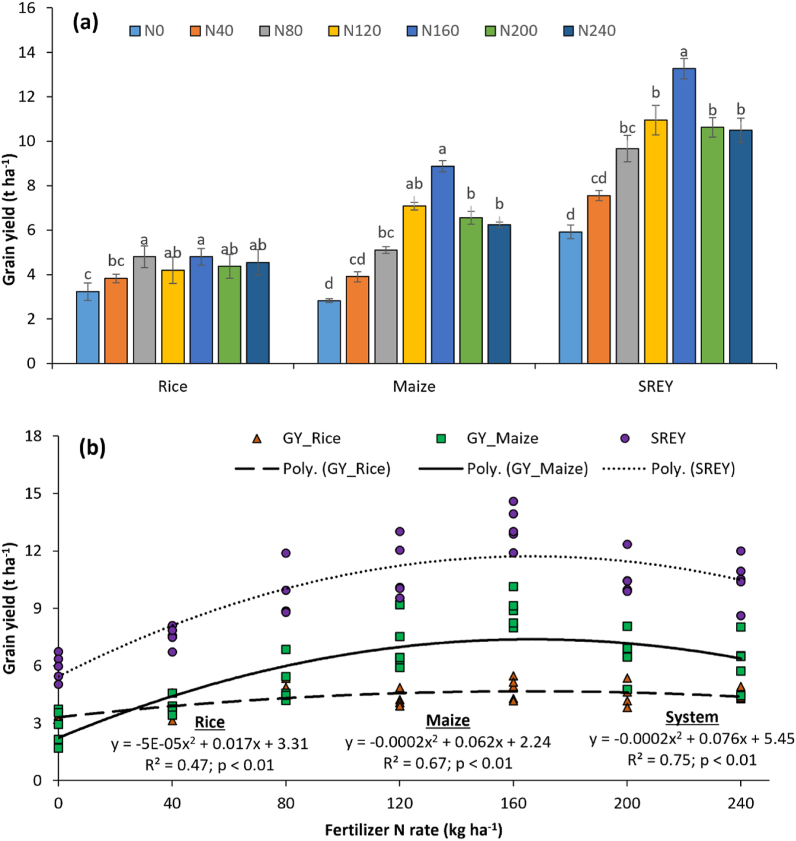
Effect of different application doses of fertilizer N on yield of rice, maize and system (a), and best fit regression to describe the interrelationship between yield and doses of fertilizer N (b). Vertical bars mean SE of means. Bars followed by at least one lowercase letter are not significantly different at p < 0.05 by Tukey's HSD test. The number after N in legends indicates kg of fertilizer N applied per ha.

The highest rice grain yield was attained in N80 and beyond that, no significant gain was noted with additional fertilizer N application (Fig. 2a). A similar trend was noted for maize yield, however, the maximum yield of 8.87 t ha^−1^ was noted in N160 followed by a decrease with additional fertilizer N application. The highest system REY of 13.3 t ha^−1^ was registered in N160 while the lowest value of 5.9 t ha^−1^ was with N0. Application of additional fertilizer N beyond 160 kg ha^−1^ could not increase the system productivity. The regression analysis was performed between yield and fertilizer N rate, and the quadratic response function (polynomial) was found to be the best fit to describe the relation (Fig. 2b). The maximum profitable fertilizer N rate was 165, 167 and 332 kg ha^−1^ for rice, maize and system, respectively.

### 3.3 Agronomic efficiency and partial factor productivity (PFP) in response to different doses of N

The agronomic efficiency of N (AEN) initially increased with an increase in the rate of fertilizer N followed by a decrease with higher doses of N (Table 2). For rice, the highest and lowest AE of N were registered for N80 and N240, respectively while these were N160 and N240 for maize. For the system, N80 recorded the highest AEN, which was at par with N40, N120 and N160 but was significantly higher (2- to 2.5-times) than N200 and N240. Unlike the AE, the PFP of N decreased gradually with an increase in the rate of fertilizer N. Maize noted a higher PFP than rice up to N80 and beyond that rice yielded higher than maize. The highest PFP of 97.5, 95.6 and 188.7 kg kg^−1^ of N were noted in N40 for rice, maize and system, respectively. The mean PFP for rice was 55.8 kg kg^−1^ of N which was higher by 28% than that of maize.

**Table 2:** Agronomic use efficiency and partial factor productivity of N as affected by different doses of N fertilizer. Mean values followed by at least one lowercase letter are not significantly different at p < 0.05 by Tukey's HSD test.

Treatments	Agronomic use efficiency (kg yield per kg N)			Partial factor productivity (kg yield per kg N)		
	Rice	Maize	System	Rice	Maize	System
N40^a^	14.9ab	50.6a	40.8ab	97.5a	95.6a	188.7a
N80	19.6a	40.3ab	46.8a	63.8b	59.9b	120.8b
N120	8.0b	43.3ab	41.9ab	59.0c	35.0b	91.2c
N160	9.8ab	43.7a	45.9a	55.5cd	30.0b	82.9c
N200	5.7b	23.4bc	23.5b	32.8d	21.8c	53.1d
N240	5.5b	18.2c	19.1b	26.0d	18.9c	43.7d
Mean	10.6	36.6	36.4	55.8	43.5	96.7

a The number after N indicates kg of fertilizer N applied per ha.

### 3.4 Plant growth responses toward different N rates

Normalized difference vegetation index (NDVI), a measure of plant vigour and plant greenness, increased gradually with the increasing rate of N application and the progress of crop season in rice. However, in the case of maize, the NDVI increased initially followed by a decrease at a higher rate of N application (Table 3). The treatment differences for NDVI were most prominent for rice. The chlorophyll content of flag leaves also registered an increasing trend with an increasing rate of fertilizer N. The N240 resulted in 1.7-, 1.7- and 1.9-times higher chlorophyll content than N0 at 39, 49 and 59 DAT, respectively in rice whereas the same was 1.2-, 1.3- and 2.8-times at 63, 73, and 82 DAS for maize. The number of tillers per hill was at par irrespective of the rate of fertilizer N at the initial growth stage (27 DAS). However, tiller numbers differed in later growth stages. At 39 DAT, N0 noted the lowest number of tillers per hill which was 22–32% lower than other treatments. No application of fertilizer N (N0) caused a reduction in plant biomass to the tune of 9–38 and 9–42% at 49 and 59 DAT, respectively. In contrast, a difference in biomass of maize was observed from the initial growth stage and no application of N fertilizer reduced the plant biomass by 5–49% than others, respectively.

**Table 3:** Different growth parameters of rice and maize as influenced by different doses of fertilizer N at different growth stages. Mean values followed by at least one lowercase letter are not significantly different at p < 0.05 by Tukey's HSD test.

Treatments	Rice				Maize				
	27 DAT	39 DAT	49 DAT	59 DAT	55 DAS	63 DAS	73 DAS	82 DAS	92 DAS
	Normalized difference vegetation index (NDVI)								
N0^a^	0.45bc	0.48d	0.61c	0.66c	0.41ns	0.51ns	0.49b	0.71c	0.67d
N40	0.42c	0.53cd	0.67b	0.69bc	0.43	0.50	0.52b	0.73bc	0.72c
N80	0.47bc	0.57c	0.72ab	0.72ab	0.39	0.51	0.53ab	0.74abc	0.75bc
N120	0.47bc	0.58c	0.70ab	0.73a	0.41	0.50	0.53ab	0.79a	0.79ab
N160	0.48bc	0.61bc	0.73ab	0.74a	0.39	0.52	0.59a	0.79ab	0.79ab
N200	0.49b	0.67ab	0.74a	0.75a	0.40	0.54	0.59a	0.79a	0.82a
N240	0.56a	0.70a	0.76a	0.74a	0.41	0.51	0.55ab	0.74bc	0.76bc
Mean	0.48	0.59	0.70	0.72	0.40	0.51	0.54	0.75	0.76
	**Chlorophyll content (mg g**^**−**^**^1^of fresh leaves)**								
N0	1.80ns	2.02c	2.31d	2.25c	1.69ns	1.21b	1.38d	0.96e	–
N40	1.76	2.56b	2.68cd	2.48c	1.80	1.24b	1.66bc	1.36d	–
N80	1.85	2.49bc	3.03bc	2.63c	1.38	1.66a	1.32d	1.79c	–
N120	1.82	2.65b	3.36b	2.58c	1.38	1.48ab	1.81b	2.04bc	–
N160	1.55	3.54a	3.33b	3.55b	1.56	1.58a	2.63a	2.15b	–
N200	1.64	3.45a	3.43ab	3.53b	1.80	1.60a	1.42cd	2.52a	–
N240	1.53	3.49a	3.91a	4.34a	1.42	1.66a	1.77b	2.68a	–
Mean	1.71	2.89	3.15	3.05	1.58	1.49	1.71	1.93	–
	***Tillers per hill***								
N0	9.7ns	10.5b	12.1b	–	–	–	–	–	–
N40	8.3	13.5a	11.7b	–	–	–	–	–	–
N80	10.0	13.9a	12.3b	–	–	–	–	–	–
N120	9.3	14.4a	15.3ab	–	–	–	–	–	–
N160	9.6	14.3a	16.8a	–	–	–	–	–	–
N200	9.7	15.4a	15.0ab	–	–	–	–	–	–
N240	8.6	14.7a	17.3a	–	–	–	–	–	–
Mean	9.3	13.8	14.3	–	–	–	–	–	–
	***Plant biomass (g)***								
N0	6.1ns	8.0ns	17.8c	17.7e	2.1b	4.4d	4.8e	10.1d	–
N40	5.5	7.1	19.6bc	19.4de	2.2ab	5.1cd	6.0de	12.2cd	–
N80	5.5	7.5	24.6ab	22.2cde	2.4ab	5.5bcd	6.8cd	12.7c	–
N120	4.9	9.3	25.2a	23.5bcd	2.6ab	5.9abcd	7.3bcd	13.4bc	–
N160	5.4	8.6	26.4a	27.1abc	2.7ab	7.0abc	8.6abc	13.9bc	–
N200	5.5	10.8	26.6a	27.9ab	2.7ab	7.3aab	9.0ab	15.1ab	–
N240	5.1	11.5	28.8a	30.7a	2.8a	8.0a	9.4a	16.5a	–
Mean	5.4	9.0	24.1	24.1	2.5	6.2	7.4	13.4	–

a The number after N indicates kg of fertilizer N applied per ha. DAT: Days after transplanting; DAS: Days after sowing.

### 3.5 Total N, total organic C and bulk density of soil under different fertilizer N application

**Fig. 3:**
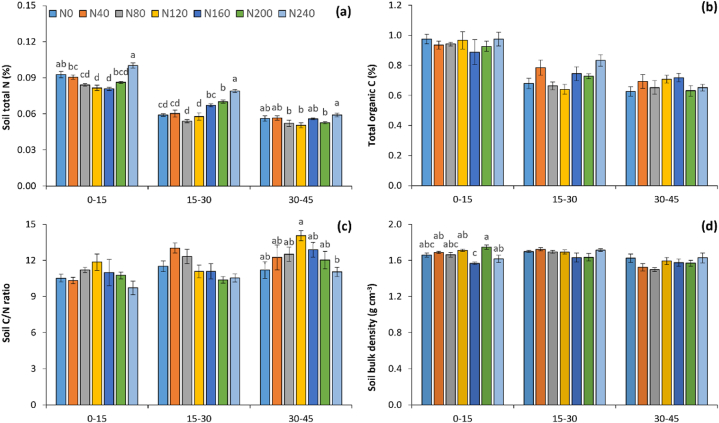
Total N (a), total organic C (b), C/N ratio (c) and bulk density (d) of soil as affected by different doses of fertilizer N. Vertical bars mean SE of means. Bars followed by at least one lowercase letter are not significantly different at p < 0.05 by Tukey's HSD test. The number after N in legends indicates kg of fertilizer N applied per ha.

Significant variations in soil total N (STN) were observed among treatments due to differential fertilizer N applications (Fig. 3a). On the surface soil (0–15 cm), the treatments which received lower (N0, N40) and higher (N240) fertilizer N recorded a comparatively higher total soil N than other treatments. The highest and the lowest STN were registered in N240 (0.10%) and N160 (0.081%). Irrespective of treatments, a decrease in STN was noted with increasing soil depth. The upper soil layer noted 13 and 60% higher STN than 15–30 and 30–45 cm, respectively. No changes in total organic C were noted irrespective of treatments and soil depths (Fig. 3b). The soil C/N ratio followed a reverse trend of STN (Fig. 3c). The soil bulk density mostly remained unchanged among treatments except for the surface layer where N160 recorded a comparatively lower bulk density (Fig. 3d).

### 3.6 Uptake and balance of N

The straw yield of rice was comparable for all treatments and ranged between 4.92 and 6.66 t ha^−1^ whereas, it differed significantly in the case of maize (Table 4). In contrast, straw N content followed an opposite trend and N content of rice straw varied significantly for rice straw while the same was at par among treatments for maize straw. The highest rice grain N uptake was noted in N240, and it was higher by 85, 49 and 26% than N0, N40 and N120, respectively. In the case of maize, N160 logged the highest N uptake of 112.7 kg ha^−1^ and beyond 160 kg N ha^−1^, the total grain uptake of N was reduced. The total plant uptake of N increased with an increased rate of N fertilizer up to 160 kg N ha^−1^ and thereafter, a reduction in uptake was observed. Application of 160 kg ha^−1^ fertilizer N increased the total plant N uptake by 146, 90, 36 and 23% in comparison to 0, 40, 80 and 120 kg N ha^−1^, respectively. The mean total N content of the 0–45 cm soil profile was 5097 kg ha^−1^ as compared to the initial stock of 5186 kg ha^−1^ which indicates a net loss of 89 kg ha^−1^ of total N from the soil profile. The highest N loss was noted for N80 and N120, and a gain in soil N stock was only noted for N240.

**Table 4:** Balance of N as affected by different doses of fertilizer N in the rice-maize system. Mean values followed by at least one lowercase letter are not significantly different at p < 0.05 by Tukey's HSD test.

Treatments	Straw/stover yield		Straw/stover N content		Grain N uptake		Straw/stover N uptake		Total plant uptake of N	Soil total N stock (0–45 cm)		Fertilizer N	Soil N balance
	Rice	Maize	Rice	Maize	Rice	Maize	Rice	Maize		Initial	Final		
	t ha^−1^		%		kg ha^−1^								
N0	4.92ns	3.73c	0.65c	0.61ns	36.6d	26.1d	55.6b	34.9c	153.2c	5186	5124b	0	−62.0ab
N40	5.82	4.64c	0.66c	0.49	45.4cd	38.2cd	68.9ab	45.7c	198.3c	5186	5112b	80	−154.1bc
N80	6.42	6.13bc	0.78b	0.48	63.2ab	58.7c	84.6ab	70.7bc	277.2b	5186	4689c	160	−656.2d
N120	5.85	7.80ab	0.80b	0.63	53.7bc	84.1b	75.4ab	92.5ab	305.6b	5186	4680c	240	−745.4d
N160	6.21	9.33a	0.88b	0.58	63.6ab	112.7a	81.8ab	118.8a	376.9a	5186	5025b	320	−480.5d
N200	6.66	6.69abc	0.85b	0.46	62.0ab	88.0ab	95.1a	89.6ab	334.8ab	5186	5157b	400	−429.1cd
N240	6.21	6.48abc	1.03a	0.58	67.7a	85.7b	92.7a	89.1ab	335.1ab	5186	5889a	480	223.1a
Mean	6.01	6.40	0.80	0.55	56.0	70.5	79.2	77.3	283.0	–	5097	–	–

#The number after N indicates kg of fertilizer N applied per ha.

### 3.7 Principal component analysis and correlation among variables

**Fig. 4:**
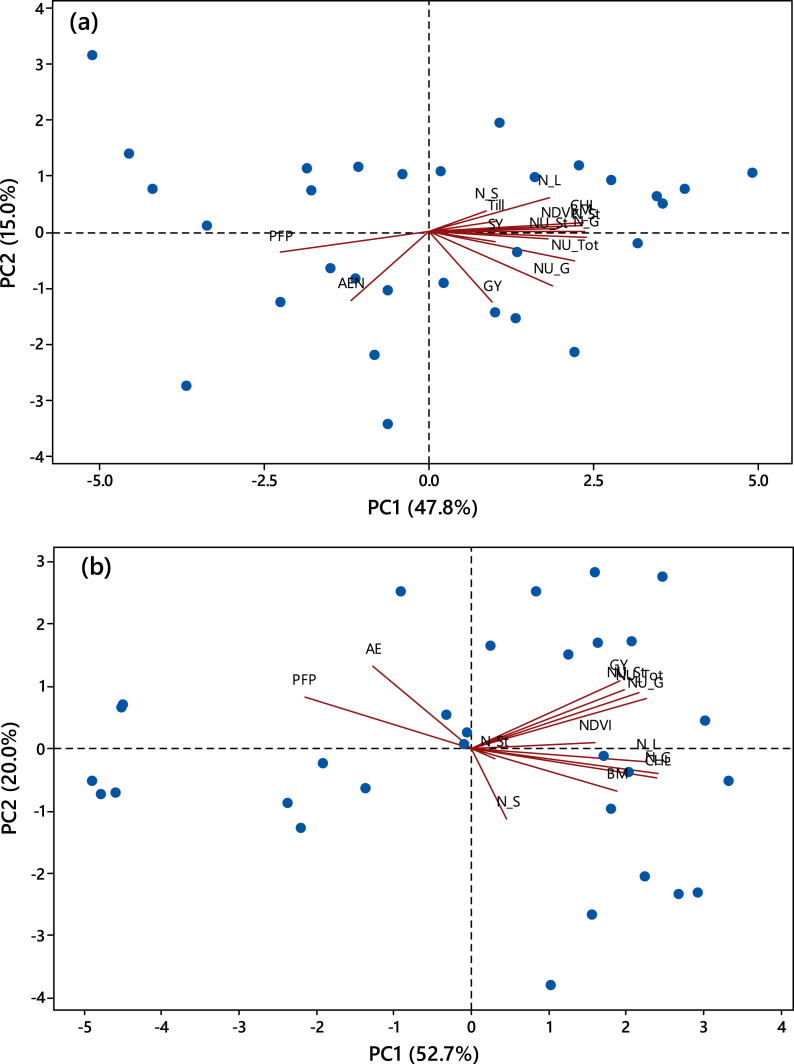
Biplots of different parameters of rice (a) and wheat (b) on principal analysis component coordinates. GY: Grain yield; SY: Straw/stover yield; N_L: Leaf total N; N_G: Grain total N; N_St: Straw/stover total N; N_S: Soil total N; NDVI: Normalized differential vegetation index; CHL: Chlorophyll; BM: Biomass; Till: Tiller number per hill; NU_G: N uptake in grain; NU_S: N uptake in straw/stover; NU_Tot: Total N uptake; AEN: Agronomic efficiency of N; PFP: Partial factor productivity of N; PC: Principal component.

The principal component analysis has revealed a good linear combination of different variables of rice and maize (Fig. 4). The first principal component (PC1) could explain 47.8 and 52.7% variability for rice and maize, respectively while the PC2 was associated with 15.0 and 20.0% variability. For rice, a close association was observed among NDVI, chlorophyll content, biomass, tiller per hill, straw nutrient uptake and grain N content (Fig. 4a). In contrast, AEN and PFP were negatively associated with PC1. A similar trend was also noted for maize (Fig. 4b). The correlation matrix also revealed significant positive correlations between different variables except for AEN and PFP which showed mostly negative correlations with most of the variables (Table 5).

**Table 5:** Pearson correlation matrix of different variables for rice and maize. ‘*’ and ‘**’ indicate statistical significance at p < 0.05 and p < 0.01, respectively.

Parameters	GY	N_L	N_G	N_St	N_S	BM	CHL	NDVI	Till	SY	NU_G	NU_St	NU_Tot	AEN
Rice														
N_L	0.200													
N_G	0.555**	0.642**												
N_St	0.585**	0.701**	0.802**											
N_S	−0.210	0.057	0.134	0.226										
BM	0.517**	0.669**	0.817**	0.839**	0.088									
CHL	0.444**	0.700**	0.833**	0.859**	0.313	0.826**								
NDVI	0.613**	0.552**	0.676**	0.700**	−0.060	0.772**	0.642**							
Till	0.408*	0.439**	0.532**	0.428*	−0.215	0.531**	0.403*	0.694**						
SY	0.230	0.558**	0.396*	0.334	−0.310	0.329	0.348*	0.365*	0.353*					
NU_G	0.930**	0.404*	0.818**	0.756**	−0.054	0.709**	0.672**	0.710**	0.503**	0.308				
NU_St	0.370*	0.690**	0.711**	0.579**	−0.164	0.581**	0.605**	0.540**	0.483**	0.924**	0.548**			
NU_Tot	0.667**	0.652**	0.851**	0.732**	−0.136	0.713**	0.713**	0.686**	0.555**	0.770**	0.520**	0.928**		
AEN	0.453*	−0.521**	−0.343	−0.431*	−0.276	−0.446*	−0.443*	−0.414*	−0.265	0.012	0.149	−0.160	−0.057	
PFP	−0.209	−0.560**	−0.682**	−0.753**	−0.186	−0.742**	−0.691**	−0.824**	−0.434*	−0.125	−0.484**	−0.390*	−0.512**	0.663**

	GY	N_L	N_G	N_St	BM	CHL	N_S	NDVI	AEN	PFP	NU_G	NU_St
Maize												
N_L	0.711**											
N_G	0.653**	0.865**										
N_St	−0.090	−0.085	−0.017									
BM	0.559**	0.739**	0.823**	−0.137								
CHL	0.679**	0.886**	0.935**	−0.095	0.851**							
N_S	−0.283	0.004	0.051	0.003	0.224	0.057						
NDVI	0.620**	0.598**	0.690**	−0.044	0.578**	0.717**	−0.293					
AEN	0.168	−0.497**	−0.632**	−0.248	−0.529**	−0.621**	−0.435*	−0.343				
PFP	−0.259	−0.771**	−0.882**	−0.218	−0.697**	−0.870**	−0.306	−0.555**	0.873**			
NU_G	0.979**	0.799**	0.785**	−0.068	0.654**	0.784**	−0.210	0.669**	−0.034	−0.451*		
NU_St	0.920**	0.649**	0.715**	−0.050	0.577**	0.690**	−0.245	0.597**	0.064	−0.340	0.932**	
NU_Tot	0.965**	0.735**	0.762**	−0.060	0.626**	0.749**	−0.232	0.643**	0.017	−0.403*	0.982**	0.983**

GY: Grain yield; SY: Straw/stover yield; N_L: Leaf total N; N_G: Grain total N; N_St: Straw/stover total N; N_S: Soil total N; NDVI: Normalized differential vegetation index; CHL: Chlorophyll; BM: Biomass; Till: Tiller number per hill; NU_G: N uptake in grain; NU_S: N uptake in straw/stover; NU_Tot: Total N uptake; AEN: Agronomic efficiency of N; PFP: Partial factor productivity of N.

## 4 Discussion

Nitrogen is the most abundantly used plant nutrient mostly applied through synthetic fertilizers. Globally, about 56% of the total fertilizer used is nitrogenous, and wheat, maize and rice are the largest consumers (∼20% each) [14]. The grain N content of rice and maize increased with increasing N application and the relationship was linear. Maize registered a higher gain in grain N content for per unit of N application than rice. The application of more nitrogenous fertilizers increases the available N content and hence, plant withdraws more N during water uptake. In contrast, no application of N caused lower N availability vis-à-vis lower uptake of N. Many researchers have also reported an increase in grain protein or N content with increasing N application in rice [22,23] and maize [24]. Mohammed et al. [25] also reported higher protein content (8.96–17.19%) with a higher dose of fertilizer N while Barraclough et al. [26] recorded an increase of 1.1–2.8% in grain N content of wheat due to higher N applications. The leaf N content also revealed a similar trend [11]. A reduction in leaf N content with increasing crop growth may be ascribed to more biomass accumulation causing dilution of leaf N.

Significant yield differences among treatments were noted among treatments for both rice and maize and ranged between 3.23-4.80 and 2.81–8.87 t ha^−1^, respectively. These yield differences can be attributed to differential fertilizer N application [27,28]. The relationship between grain yield and fertilizer N could best be explained by quadratic relationships indicating an economic optimum for fertilizer, beyond which the cost of additional fertilizer offsets the yield gain [[29], [30], [31]]. The economic optimum dose for our case was 165 and 167 kg ha^−1^ for rice and maize, respectively. Kumari et al. [32] reported 160–200 kg N ha^−1^ to be optimal for maize under limited and adequate irrigation. Likewise, Chen et al. [33] reported 160 kg N ha^−1^ as the economic optimum dose of N for maize under rainfed conditions in northeast China plain and are in good agreement with our findings. Ju and Christie [34] and Zhao et al. [35] reported an initial increase in rice yield followed by a decrease with the application of the increasing amount of fertilizer N. A lower rice yield under high fertilizer N application could be due to a higher N content of the plant tissue that augments N metabolism vis-à-vis carbohydrate consumption causing poor carbohydrate translocation during grain filling [36]. A reduction in grain filling rate, number of filled grains and test weight at higher N applications were responsible for lower yield at higher fertilizer doses [35]. Moreover, application of N at increased rates increases the number of spikelets per m^2^ and prolonged the grain filling stage causing low and poor grain filling. The maize was more responsive than rice and recorded a 62 kg increase in grain yield per kg of fertilizer N applied while the same was only 17 kg for rice. The absence of fertilizer N resulted in higher grain yield in rice (3.23 t ha^−1^) than in maize (2.81 t ha^−1^). This could be ascribed to a relatively lower N demand of rice than maize, and the soil reserve N was more available to rice as it was cultivated before maize. Moreover, maize requires higher soil fertility or more N input for maximal yield [37,38]. Halvorson et al. [39] Ma et al. [40] and Biswas and Ma [41] reported an increase in maize grain yield with an increasing N rate up to 224, 120 and 150–200 kg ha^−1^, respectively. An improvement in yield and yield parameters of maize has also been recorded by other researchers [32,[42], [43], [44], [45]].

The key variables shaping N utilization are grain yield, percent of grain N and total N uptake. Under similar N application, a higher grain N content can only be achieved at the expense of lower grain yield or by higher N uptake or greater translocation efficiency from leaf to grain. Higher agronomic use efficiency of N was achieved at 80 and 160 kg N ha^−1^ for rice and maize, respectively and beyond that AUE decreased with increased use of fertilizer N. Similar findings have also been reported by many researchers [41,46]. Unlike AUE, the PFP gradually diminished with the higher application of fertilizer N and a steeper decrease in PFP was visible for maize than rice. The trend of NUE was similar to AUE and the mean NUE was 0.42 and 0.75 for rice and maize, respectively. A higher NUE in maize could be explained by the presence of aerobic conditions during the growth period minimizing loss of N either through leaching or volatilization. In contrast, anaerobic conditions due to stagnant water during rice cultivation augmented N loss mostly through volatilization and vaguely through leaching. N factor productivity, N use efficiency and recovery of N in grain were positively correlated with the grain yield as previously reported by Raun and Johnson, and López-Bellido and López-Bellido [18,47].

Crops rely on the available mineralizable N in the soil for growth [48]. Plant vigour which is indicated by NDVI among many was more when higher doses of fertilizer N were applied [49]. The chlorophyll content of leaves, tiller number (for rice) and plant biomass also recorded a similar trend, particularly at the later crop growth stage. Abdou et al. [27] also reported better growth parameters at higher N applications. At the initial growth stage, the N requirement was lower and soil N along with applied fertilizer N were sufficient to meet the crop demand. However, as the crop season progressed, the available N was not sufficient to fulfil the N demand resulting in lower chlorophyll content, biomass yield and lesser tiller (for rice). Studies revealed a direct positive correlation between fertilizer N application and total chlorophyll content of leaves [50]. The effect of differential N application was most prominent in later growth stages. Nitrogen is an important component of protein and nucleic acids and therefore, essential for every cell formation [51]. It is also the most important constituent of chlorophyll that is responsible for the synthesis of carbohydrates. Hence, inadequate N availability affects chlorophyll formation and ultimately, hinders the biomass accumulation of plants [38,[52], [53], [54]]. Hence, the supply of N needs to be ensured for achieving potential yield [55].

A differential uptake pattern of soil N was evident from the STN graph. Higher STN in lower fertilizer N treatments could be ascribed to no or low application rate of fertilizer N followed by poor crop growth as evident from different plant parameters while the same for higher fertilizer N applied treatments could be due to higher doses of N application. In contrast, the treatments which received fertilizer doses of 120–200 kg N ha^−1^ recorded lower STN content and this could be attributed to more vigorous crop growth resulting in a depletion of STN status.

A similar straw yield but significantly different straw N content indicates a dilution of nitrogen in rice straw under lower N application. However, the trend was the opposite in maize and stover N content was similar but stover yield differed significantly among various treatments of fertilizer N. However, Biswas and Ma [41] reported no change in stover yield due to different N applications in maize. A higher biomass yield was associated with higher fertilizer N application and similar findings were reported by other researchers [41,56]. Plant N accumulation increased with higher N application [56]. The plant uptake of N was augmented with increased N application throughout the range in rice but for maize, the maximum N uptake was noted for 160 kg N ha^−1^. The soil N stock which was calculated on an equal soil mass basis for a 45-cm soil profile showed a net loss in total N at a lower N application rate. Maximum depletion in total soil N was noted for 160 kg N ha^−1^ followed by 120 kg N ha^−1^. A positive balance i.e., a gain in total soil N was only noted for 240 kg N ha^−1^, indicating the applied fertilizer N was more than sufficient to meet the crop demand. A portion of the N-input never became available to the crop and may have been lost from the soil mineral N pool and or the soil-plant system [57].

## 5 Conclusion

Higher production of high-quality grain (higher grain N content) requires higher application of fertilizer and greater uptake by crops. Therefore, the important challenge to the researchers is to increase the productivity of crops with better grain quality for sustainably achieving food and nutritional security. We have observed, an increase in grain yield in rice and maize up to 160 kg ha^−1^ and beyond that, no gain in grain yield was noted. The economic optimum of fertilizer N for the rice-maize cropping system was 332 kg ha^−1^ for rice-maize cropping system. The grain N content increased with N-application rates suggesting a way to improve the grain quality in terms of protein content. However, the optimum dose of fertilizer N for getting better grain quality needs to be worked out through future research. A decrease in yield at higher N doses suggests the negative impact of fertilizer N when over-applied. Hence, fertilizer level must be optimized to balance grain yield, grain N content and maximal profit.

## Author contribution statement

Surajit Mondal: Conceived and designed the experiments; Performed the experiments, Analyzed and interpreted the data; Contributed reagents, materials, analysis tools or data; Wrote the paper.

Rakesh Kumar: Performed the experiments; Analyzed and interpreted the data; Wrote the paper.

Janki Sharan Mishra: Conceived and designed the experiments; Wrote the paper.

Sanjeev Kumar: Analyzed and interpreted the data; Wrote the paper.

Vinod Kumar Singh: Conceived and designed the experiments; Contributed reagents, materials, analysis tools or data; Wrote the paper.

Kumar Varun Vijay, Manisha Kumari and Sana Raza Khan: Performed the experiments; Wrote the paper.

## Data availability statement

Data included in article/supplementary material/referenced in the article.

## Declaration of competing interest

The authors declare that they have no known competing financial interests or personal relationships that could have appeared to influence the work reported in this paper.

## Abbreviations

AE: Agronomic efficiency of nitrogen
DAS: Days after sowing
DAT: Days after transplanting
MSP: Minimum support price
N: Nitrogen
NDVI: Normalized difference vegetation index
NH_3_: Ammonia
NUE: Nitrogen use efficiency
PC: Principal component
PFP: Partial factor productivity
REY: Rice equivalent yield
STN: Soil total nitrogen
